# Safety and efficacy of transurethral laser lithotripsy and percutaneous laser lithotripsy in 41 dogs with lower urinary tract stones

**DOI:** 10.3389/fvets.2025.1643635

**Published:** 2025-10-28

**Authors:** Jin Shigemoto, Mitsunobu Kawazu

**Affiliations:** Oji Pet Clinic-Tokyo Animal Minimally Invasive Medical Center, Tokyo, Japan

**Keywords:** canine, urinary tract stones, transurethral, laser lithotripsy, holmium:YAG, percutaneous, minimally invasive surgery

## Abstract

**Objective:**

The clinical application of transurethral laser lithotripsy (TUL) for lower urinary tract stone removal in dogs is constrained by factors such as body weight, stone size, and stone number. This study evaluated the safety and efficacy of TUL and percutaneous laser lithotripsy (PL) in cases where TUL alone was not feasible.

**Study design:**

Retrospective study.

**Animal population:**

Forty-one dogs (24 males, 17 females) were included between June 15, 2017, and January 26, 2023. Among them, 13 males were castrated and 14 females spayed.

**Method:**

TUL was performed using a holmium:yttrium-aluminum-garnet (Ho:YAG) laser, an 8.5Fr flexible ureteroscope, and a 9.5Fr rigid cystoscope for urethral and bladder stone fragmentation. PL was conducted using a Ho:YAG laser in combination with percutaneous cystolithotomy (PCCL).

**Results:**

TUL was performed in 34 dogs, including 22 males (64.7%) and 12 females (35.2%). Laser lithotripsy was categorized by endoscope type and stone location. Of these, 33 dogs (94.1%) completed the procedure, while one male (2.9%) required conversion due to excessive bleeding. PL was performed in seven dogs (two males, 28.5%; five females, 71.4%), all of whom (100%) completed the procedure without conversion. Complications from laser lithotripsy occurred in five males (12.1%) of 41 dogs.

**Conclusion:**

TUL is a minimally invasive urethral procedure, but its feasibility is limited in underweight dogs where endoscope insertion is impractical.

**Clinical significance:**

When TUL alone is unviable, combining it with PL provides a safe and effective laser lithotripsy approach for bladder and urethral stones, regardless of the dog's weight or sex.

## 1 Introduction

Urolithiasis is a common urologic disease in dogs and cats, leading to recurrent hematuria, urinary tract infections, obstruction, acute kidney injury, and sometimes death. Diagnosis includes urinalysis, urine culture, blood tests, radiography, and stone composition analysis, followed by medical or surgical intervention (1–4). Medical treatment focuses on stone dissolution, with dietary management aimed at regulating urinary salt concentrations and increasing water intake to reduce urinary supersaturation (5–7), a key factor in stone and crystal formation. Surgical removal is preferred for stones that cannot be dissolved. Traditionally, laparotomy has been used (8, 9), but minimally invasive techniques such as percutaneous cystolithotomy (PCCL), extracorporeal shock wave lithotripsy (ESWL), and laser lithotripsy have recently been reported. In dogs, PCCL has been associated with significantly shorter hospital stays than open cystotomy, with no difference in operative time (10–26). Several reports describe the use of ESWL, which applies externally generated shock waves to fragment kidney and ureteral stones in animals. However, its application may be limited by high costs, restricted availability of the lithotripsy device, patient size, ureteral diameter, and stone size (15–18).

Laser lithotripsy is an innovative technique that employs an endoscope to fragment and facilitate the expulsion of urinary tract stones. While widely used in humans as a standard treatment for urolithiasis, its application in animals remains less common (27–29). Several reports describe holmium:yttrium-aluminum-garnet (Ho:YAG) laser lithotripsy as an effective stone treatment in animals, including goats and horses. These studies highlight its use as a minimally invasive approach for lower urinary tract stones, avoiding the need for bladder or urethral incisions (11, 30). Transurethral laser lithotripsy (TUL) is a minimally invasive and effective surgical method; however, its feasibility in dogs is restricted by urethral size, particularly in males, necessitating the use of a flexible ureteroscope. Although TUL is less invasive than conventional surgical procedures, it may have prolonged operative times. If excessive bleeding or poor visibility occurs, conversion to PCCL or laparotomy is required. Additionally, when bladder stones are large or numerous, fragmentation and dusting may be time-consuming, making conventional laparotomy a more appropriate option (22–24). Even in cases with anatomical restrictions, laser lithotripsy may still be feasible through percutaneous laser lithotripsy (PL), which combines percutaneous bladder stone removal with laser lithotripsy. This approach is particularly beneficial for dogs with large or multiple bladder stones where TUL is impractical. This study aimed to retrospectively evaluate the effectiveness and safety of TUL and PL in dogs with bladder and ureteral stones.

## 2 Materials and methods

### 2.1 Criteria for case selection

This retrospective study included 41 dogs that underwent laser lithotripsy at Oji Pet Clinic between June 15, 2017, and January 26, 2023, comprising 34 cases of TUL and seven cases of PL. TUL inclusion criteria were male dogs with a perineal urethral fistula, those where an 8Fr catheter could be inserted, and those weighing ≥5 kg. Female dogs weighing < 5 kg were excluded due to the expected difficulty in rigid cystoscope insertion. PL had no weight restrictions. Dogs included in the study exhibited clinical signs, had uroliths that could not be spontaneously expelled, or had partial or complete urethral obstruction requiring TUL or PL. The surgical method was selected based on anatomical feasibility. TUL was performed in males using an 8.5Fr flexible ureteroscope and in females using a 9.5Fr rigid cystoscope. A final test to check for successful catheter insertion was performed under general anesthesia. If a flexible ureteroscope could not be advanced or if bladder stones were large or numerous, PL or PCCL was performed. X-ray and abdominal ultrasonography were used to assess surgical eligibility, factoring in stone size and location.

In male dogs, TUL was indicated for five or fewer urethral stones. For bladder stones, TUL was performed if a single stone measured ≤ 20 mm or if two stones were ≤ 10 mm. In female dogs, TUL was performed for up to two bladder stones ≤ 20 mm or up to five stones ≤ 10 mm.

If excessive bleeding or poor visibility occurred, the procedure was converted to PL or PCCL. Owners were informed that multiple laser lithotripsy sessions might be necessary and that residual sand-like stone fragments could remain postoperatively. Informed consent was obtained before surgery. Before laser lithotripsy, all dogs underwent a physical exam, complete blood count, blood chemistry panel, abdominal ultrasonography, coagulation profile, and electrocardiography. A procedure was deemed unsuccessful if it could not be completed or if intraoperative bladder damage was observed. However, minor postoperative bladder irritation from surgical irrigation was not considered a procedural failure.

### 2.2 Method of laser lithotripsy

**Figure 1:**
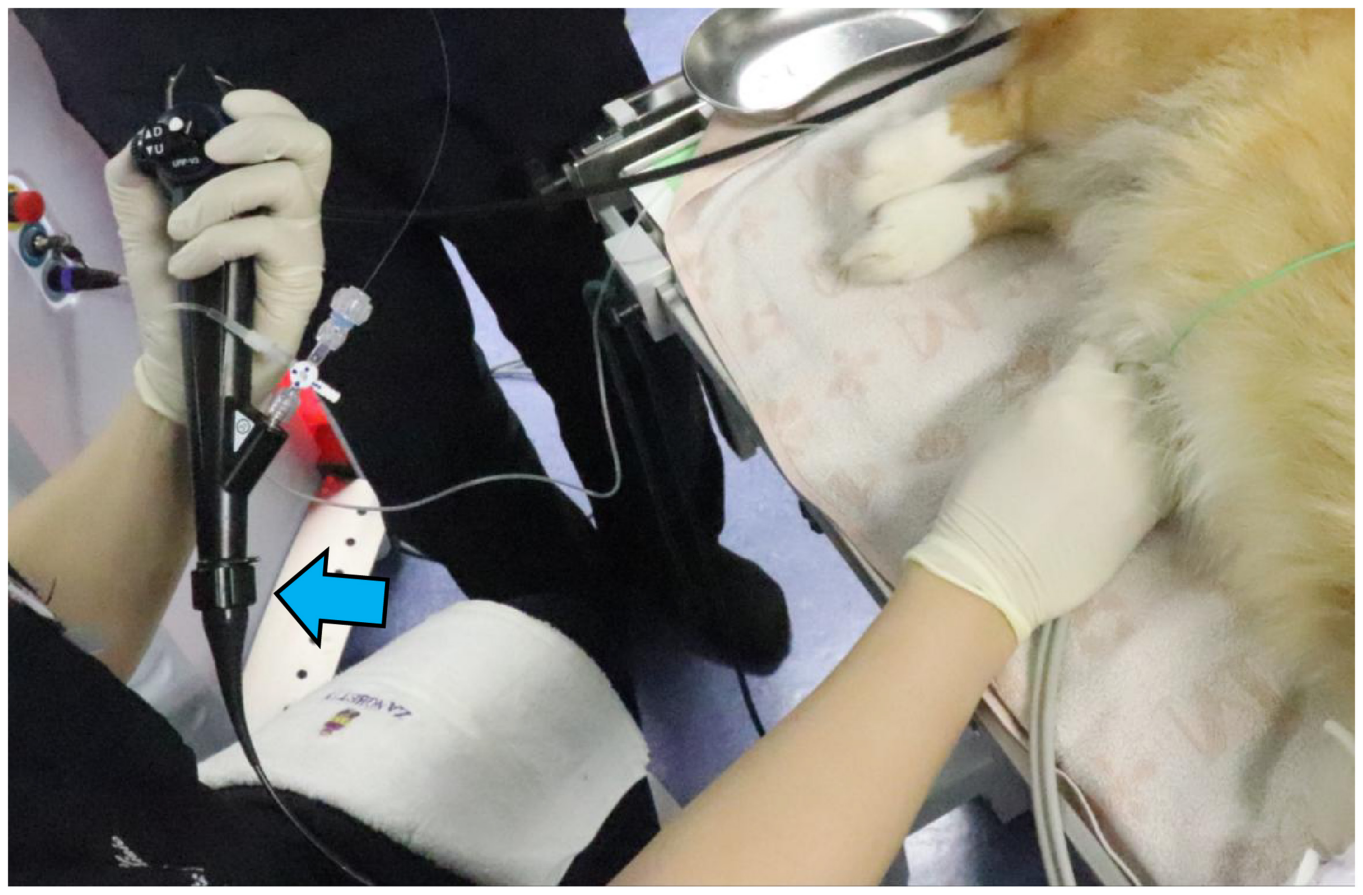
Photograph of a male dog undergoing TUL. The dog had a complete blockage of the urethra due to a stone; therefore, UUL was performed. A flexible ureteroscope (blue arrow) was inserted, and fragmentation was performed.

**Figure 2:**
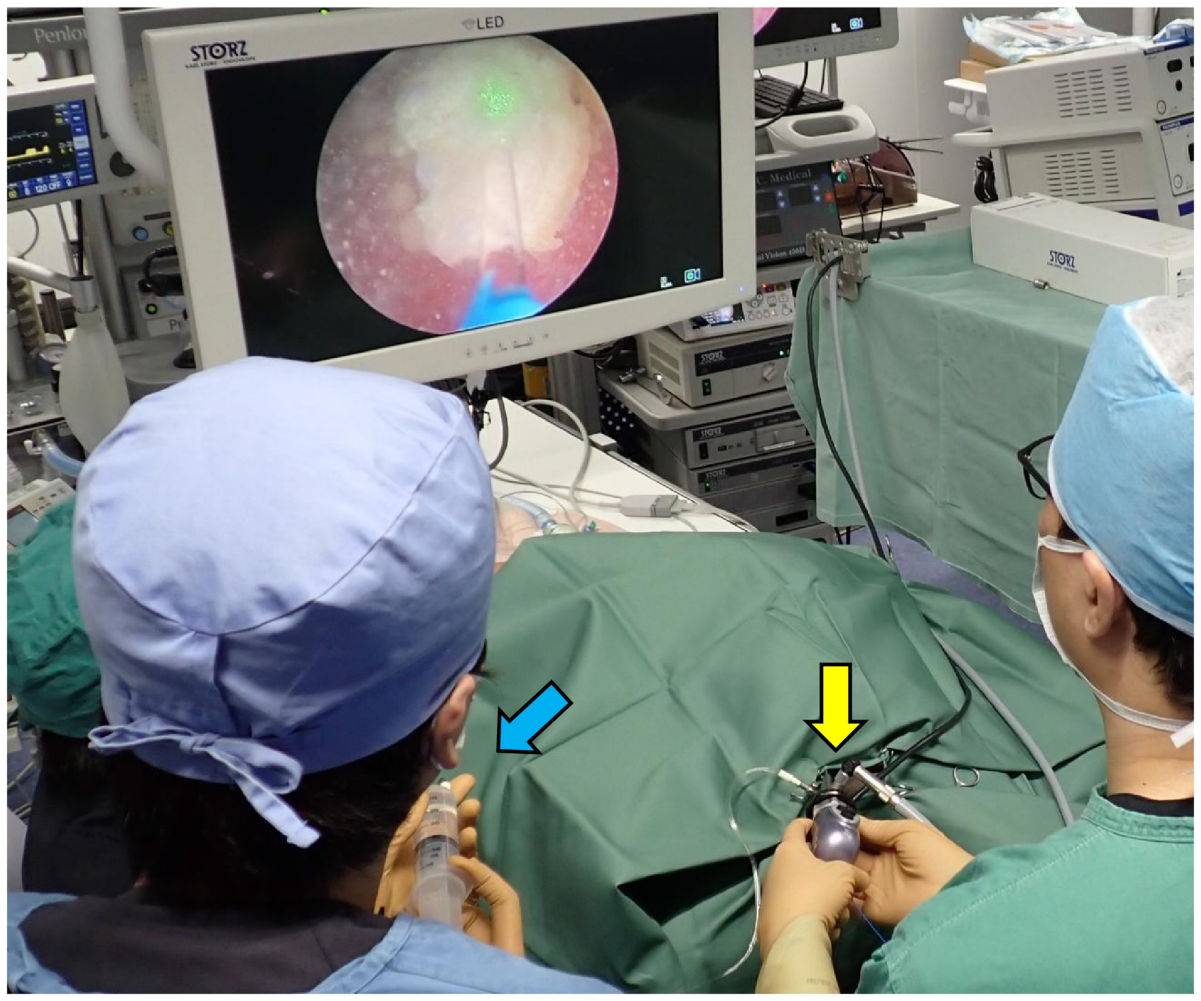
Photograph of a female TUL during REC. The surgeon is operating the rigid cystoscope (yellow arrow), and the assistant is injecting saline (blue arrow).

For TUL, male dogs were positioned in left lateral recumbency. The foreskin was shaved, and the inner prepuce was thoroughly washed with saline. The surrounding area was disinfected with 0.5% chlorhexidine alcohol before shaving the external genital region. A flexible ureteroscope (FLEX-XC 67030BA; Karl Stortz, Tuttlingen, Germany) was used for stone fragmentation and dusting by a single surgeon (Figure 1). Saline irrigation was continuously administered by an assistant, while another assistant managed fragment collection using a TIPLESS stone basket (Stone basket TIPLESS; UROTECH, Rohdorf, Germany). A second assistant assisted with palpation and endoscope insertion to prevent excessive bladder distension. Saline irrigation was continuously administered during stone fragmentation, with flow manually adjusted to maintain clear visualization. To prevent fragments from striking the endoscope, saline was intermittently injected during fragmentation and dusting. The bladder was palpated, and when it became tense, saline was aspirated. Fragmentation was paused to avoid fragment impact on the endoscope tip during suction. Fragment size was determined by crushing the largest visible fragment to a size suitable for basket retrieval. After fragmentation, blood clots and fine particles were removed via bladder lavage using an 8Fr catheter. For female TUL, patients were positioned in the right or left lateral recumbency position, and the perivulvar area was trimmed and disinfected with 0.5% chlorhexidine alcohol before sterile draping. A single operator controlled the endoscope and performed fragmentation (Figure 2), while an assistant managed irrigation and another handled basket forceps for stone retrieval. A dedicated assistant performed palpation to prevent excessive bladder tension. Stones were fragmented while saline was injected to protect the rigid cystoscope (Operating Telescope 30°67030BA; Karl Stortz) from damage. The largest fragments were further reduced for basket retrieval. In cases where the rigid cystoscope could not access the bladder in large females, a flexible ureteroscope was used instead.

**Figure 3:**
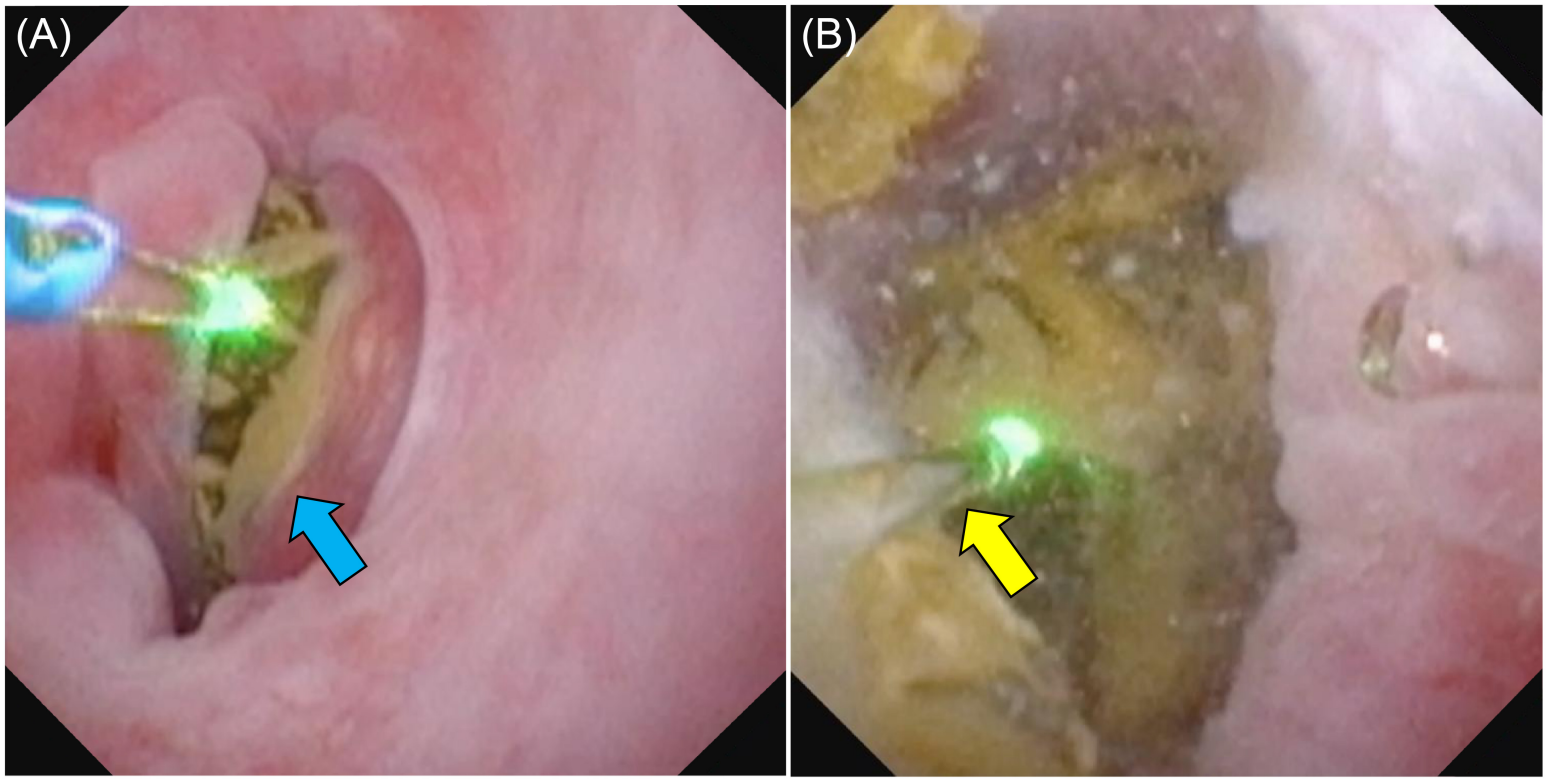
Photograph of a male dog undergoing UUL. (**A**) The urethral calculus (blue arrow) was completely blocking the dog's urethra. (**B**) As the calculus was completely stuck, we used saline solution to clear the field of view and a laser to break up the calculus (yellow arrow). The broken calculus was collected using basket forceps.

**Figure 4:**
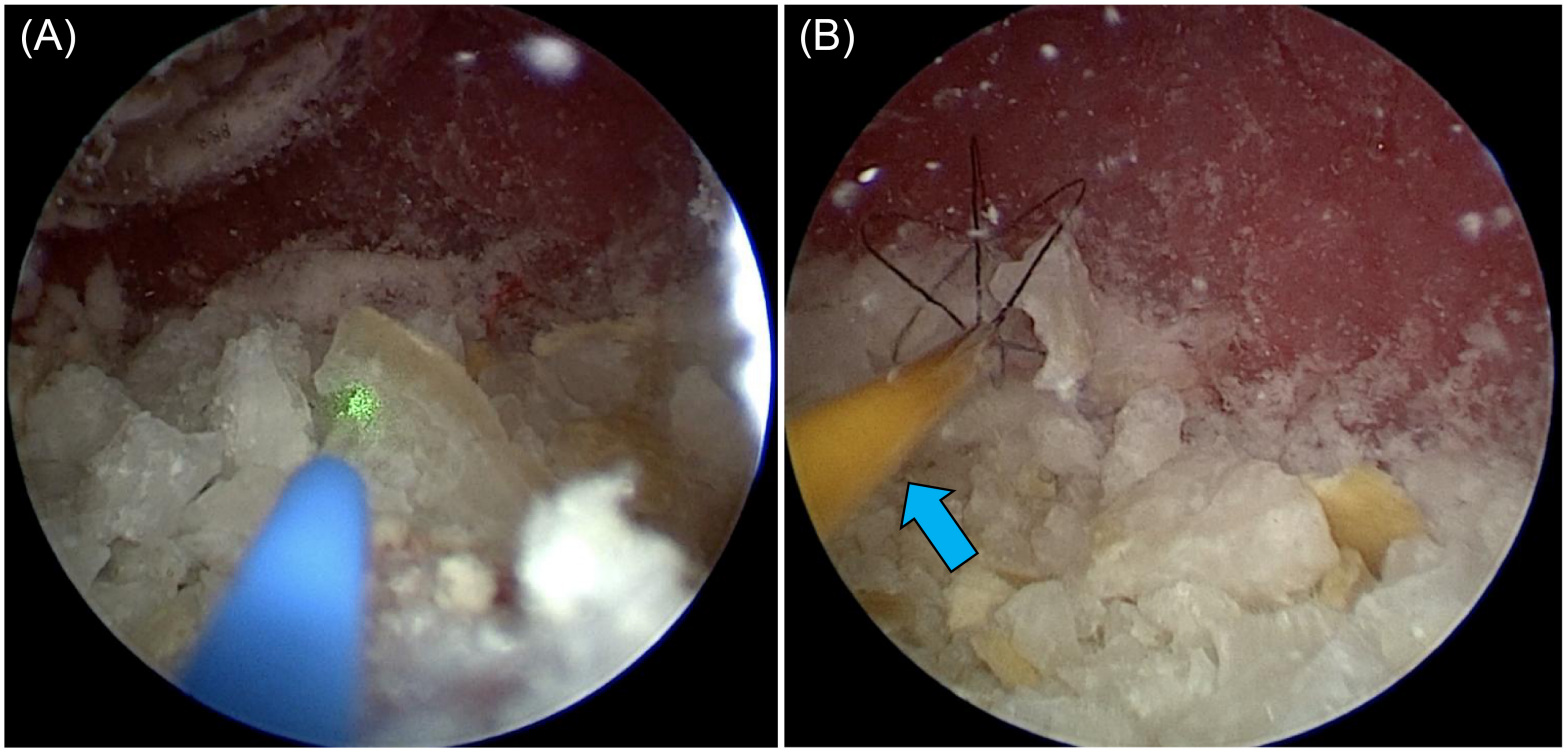
Female dog undergoing REC. (**A**) A laser was used to break up the stones in the bladder. (**B**) The larger fragments were collected using basket forceps (blue arrow).

TUL is a general term for techniques involving endoscopic insertion through the urethra. It includes ureteroscope urethral lithotripsy (UUL) (Figure 3), ureteroscope bladder lithotripsy (UBL), ureteroscope urethral-bladder lithotripsy (UUBL), and rigid cystoscopic lithotripsy (RCL) (Figure 4). UUL is a surgical procedure that employs a flexible ureteroscope to fragment urethral stones. UBL is a surgical procedure that employs a flexible ureteroscope to fragment bladder stones. UUBL is a surgical procedure that employs a flexible ureteroscope to fragment both urethral and bladder stones. RCL is a surgical procedure that employs a rigid cystoscope to fragment bladder stones. Classification was based on the endoscope type and lithotripsy site.

**Figure 5:**
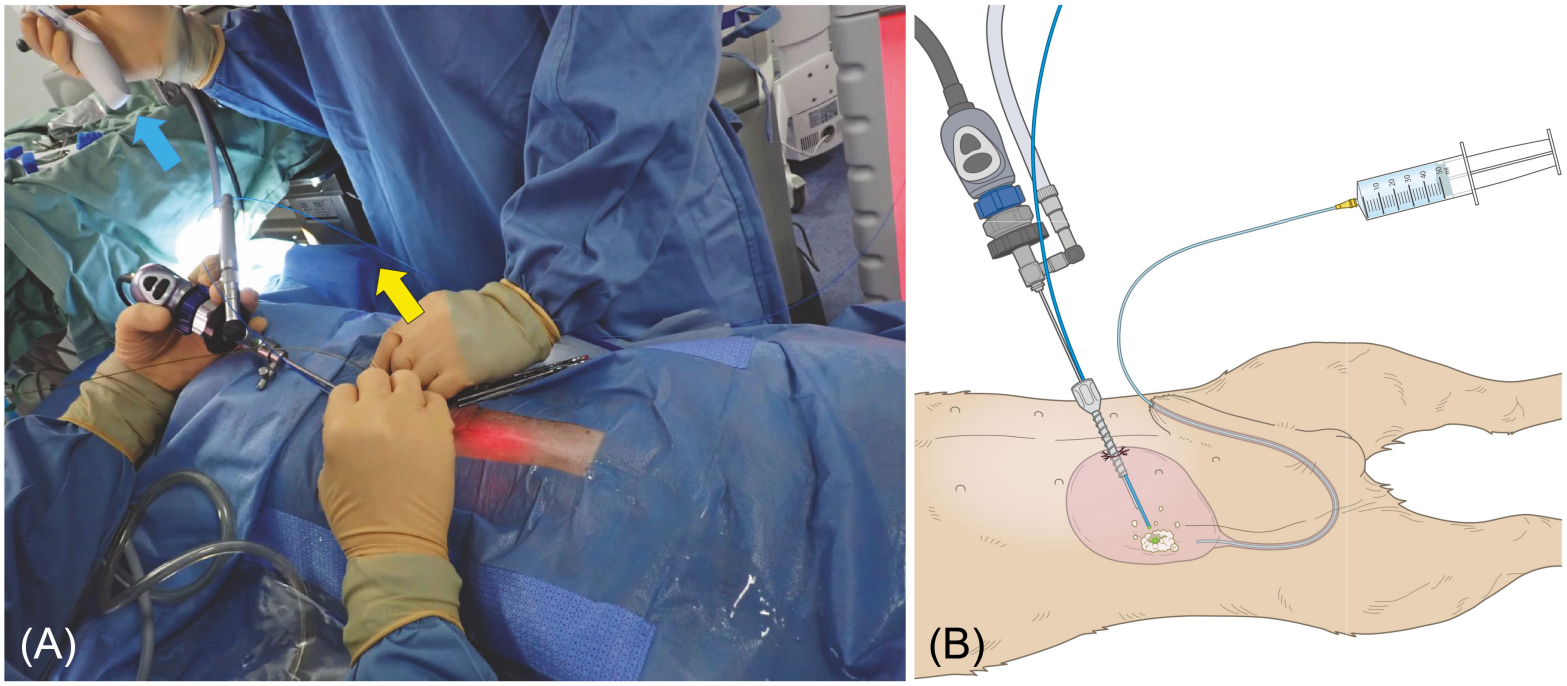
(**A**) Female dog undergoing PL. The stone was grasped with a basket forceps (blue arrow) and crushed with a laser fiber (yellow arrow). (**B**) Male dog undergoing PL. The image is of a male dog with a clearly visible urethra to make the diagram easier to understand. The bladder is inflated with a saline solution via a catheter. The bladder is then palpated and sutured to the skin percutaneously. The diagram shows the trocar being inserted into the visible bladder and the laser being used to fragment the tissue finely.

**Figure 6:**
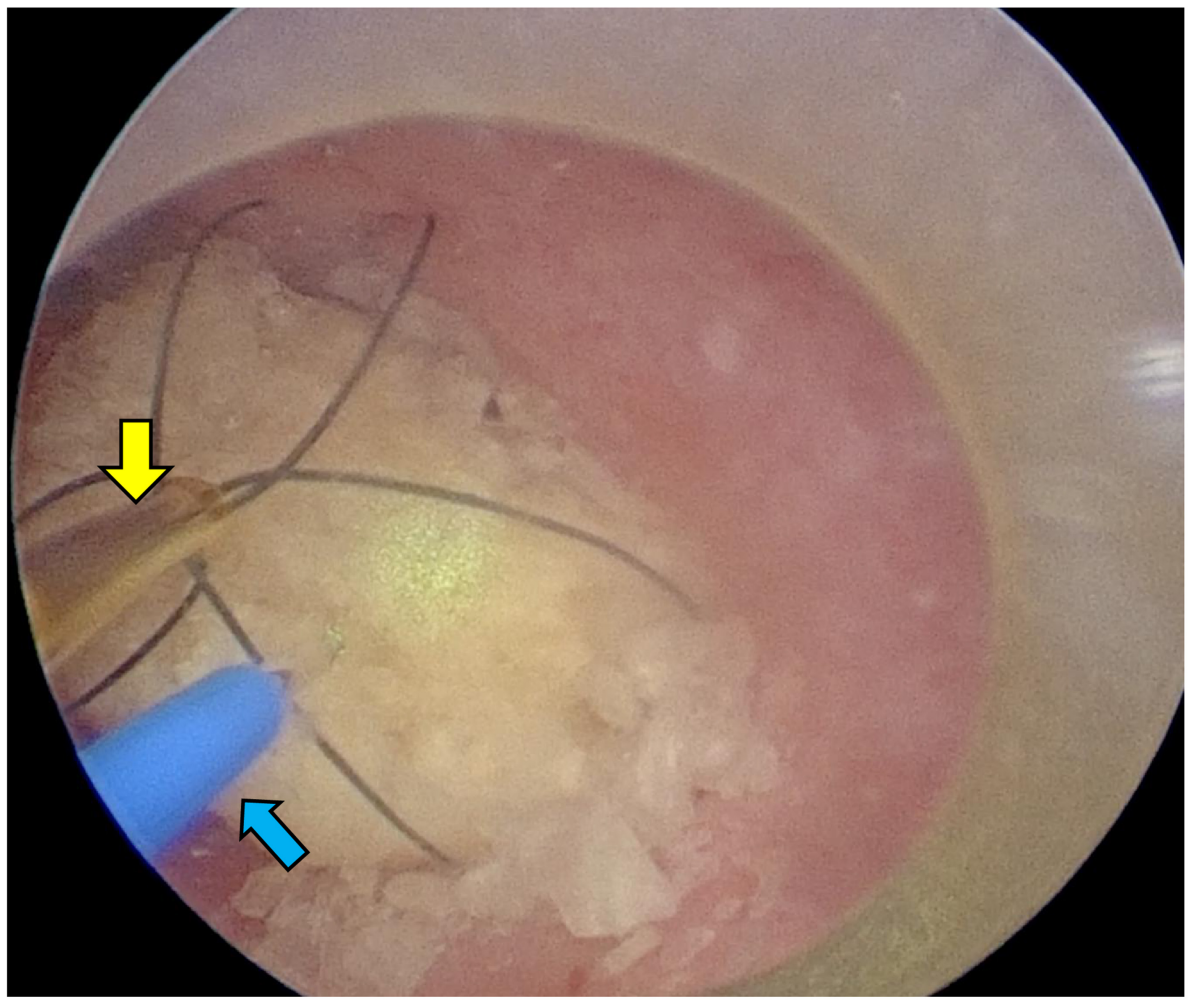
Female dog undergoing PL. The laser (blue arrow) was inserted after grasping it with a basket forceps (yellow arrow) through a trocar, and it was fragmented to a size that could be recovered.

After shaving and disinfecting the abdomen, the PCCL site was isolated with a sterile drape. A catheter was placed in the urethra, and saline was injected into the bladder. Once palpable, the bladder was secured to the skin with 5–6 simple ligatures. A 6 mm port (cannula, size 6 mm 60120MS; Karl Storz) was inserted following a 3–5 mm bladder incision using a No. 11 scalpel. Saline was injected through the urethral catheter to inflate the bladder and expand the field of view. A 2.7 mm 0° telescope (Hopkins II Telescope 7220AA; Karl Storz) and laser fiber were inserted into the cannula simultaneously, with stones fragmenting while saline was injected through the urethral catheter (Figures 5A, B). Fragments and smaller stones were flushed out through the trocar, whereas larger stones were retrieved using a basket (Ultra-Catch NT6wire; Olympus, Tokyo, Japan). Stones too large for PCCL extraction were treated with PL combined with laser lithotripsy (Figure 6).

In all dogs, laser lithotripsy was performed using a Ho:YAG laser (Sphynx Jr; LISA Laser Products, Kathenburg-Lindau, Germany) with a frequency of 1–25 Hz, pulse energy of 0.3–3.5 J, pulse width of 100–650 μs, and maximum output of 18 kW. Laser lithotripsy was performed using either 1.0 J, 10 Hz, EFFECT50, or 1.1 J, 7 Hz, EFFECT30. A 272-micron laser fiber was used with flexible ureteroscopes, and a 365-micron fiber with rigid cystoscopes. While a 365-micron fiber is technically compatible with flexible ureteroscopes, the unique anatomy of animals, specifically the presence of a penile bone, often introduces significant curvature to the urethra. This curvature can hinder the flexible ureteroscope's maneuverability, especially with the larger 365-micron fiber. Consequently, based on our experience and subjective assessment of ease of operation in this anatomical context, we opted for the 272-micron laser fiber to optimize procedural control. Finally, to check for residual stones, an X-ray analysis was performed to confirm that no obvious stones >3 mm were present. For stones that would not appear on an X-ray, an ultrasound was used. Fragmentation success was determined by: (1) fragment size ≤ 3 mm on X-ray; (2) endoscopic visualization and capture of the largest fragments; and (3) unobstructed passage of basket-retrieved fragments through the urethra. To achieve this, we performed bladder irrigation immediately after surgery, ensured the dog was standing and urinating properly, and confirmed that no residual stones >3 mm appeared on an X-ray and abdominal ultrasound before surgery completion. The stones were analyzed by infrared absorption spectrophotometry. Urine cultures were performed in-house using the disk diffusion method to determine atntimicrobial susceptibility (amoxicillin, amoxicillin-clavulanate, cephalexin, chloramphenicol, doxycycline, enrofloxacin, faropenem, gentamicin, and minocycline). In cases where no antibiotics were predicted to be effective based on susceptibility results, bacterial cultures and susceptibility testing were outsourced to external laboratories. In addition, we strengthened postoperative dietary and medical treatment to prevent urinary tract stones.

### 2.3 Anesthesia

To prepare for anesthesia, dogs scheduled for non-emergency procedures were fasted for 12 h and water-deprived for 6 h. Premedication included midazolam (0.2 mg/kg IV or subcutaneously; Dormicum; Astellas Pharma Inc., Tokyo, Japan), buprenorphine (0.3 mg/kg), and atropine sulfate (0.01 mg/kg IV or subcutaneously; Atoropin; Nipro ES Pharma Co., Ltd., Osaka, Japan), administered IV or SC. Anesthesia was induced with propofol (6–10 mg/kg IV; propofol 1%; Intervet K.K., Tokyo, Japan) and maintained with isoflurane (Isoflu; DS Pharma Animal Health Co., Ltd., Osaka, Japan) or sevoflurane (Sevofrane; Pfizer Japan Inc., Tokyo, Japan), as needed to maintain a mean arterial pressure ≥60 mmHg. IV fluid therapy was maintained with lactated Ringer's solution at 5–10 ml/kg during the procedure.

## 3 Results

### 3.1 Case information

Overall, 24 males and 17 females were included in this study, of which 13 were castrated males and 14 were spayed females. They included the following breeds: six Toy Poodles, five Miniature Schnauzers, five mixed breeds, two Norfolk Terriers, two Chihuahuas, two Yorkshire Terriers, two Pekingese, one Shih Tzu, one American Cocker Spaniel, one French Bulldog, one Miniature Pinscher, one Papillon, one Shiba Inu, one Shetland Sheepdog, one Norwich Terrier, one Welsh Corgi, one Kai Inu, one Pug, one Bulldog, one Rottweiler, one Jack Russell Terrier, one Cavalier King Charles Spaniel, one Labrador Retriever, and one Newfoundland.

The main sign was difficulty urinating due to urethral stones in 14 male dogs. Nineteen dogs had both stranguria and hematuria, seven had hematuria only, and one had no signs. The median age was 93 (11–178) months, and the median weight was 6.7 (2.28–49.2) kg.

### 3.2 UUL cases

In total, eight of 24 male dogs were selected as UUL, with all dogs having urethral stones and obstruction. The stones were fragmented and collected using a flexible ureteroscope, and the operation time was defined as the time from the insertion of the flexible ureteroscope to the recovery of the fragmented stones. The median operation time was 66 (25–126) min, and body weight was 5.1 (3.6–39.9) kg (Table 1). Of the eight dogs, only one (12.5%) had urethral stones < 3 mm, six of eight dogs (75%) had urethral stones between 3 and 6 mm, and one of eight (12.5%) had urethral stones between 6 and 9 mm. Three of eight dogs (37.5%) had one urethral stone, and five (62.5%) had multiple urethral stones (up to 5). The fragments were collected in a basket, and as many of the dogs as possible were made to urinate while standing to flush out any remaining small stones. UUL was completed in all eight cases (100%), and the complications were testicle swelling, perineal edema in one case (12.5%), and one dog (12.5%) developed urethral stricture and required perineal urethrostomy after 3 months.

**Table 1:** The site where laser lithotripsy was performed, the dog's sex and weight, the duration of the surgery, and complications.

**Location of laser lithotripsy**	**Sex (M/F)**	**BW (kg)**	**Operating time (min)**	**Complications**
TUL:UUL	M: 8	5.1 (3.6–39.9)	66 (25–126)	Edema and inflammation of the testicular foreskin: 1
TUL:UUBL	M: 3	5.1 (4.5–5.2)	129 (90–236)	Conversion to PCCL due to bleeding: 1
				Bladder injury due to postoperative bladder leakage: 2
				Edema and inflammation of the testicular foreskin: 1
TUL:UBL	M: 11, F: 1	13.1 (3.0–49.2)	103 (56–230)	Edema and inflammation of the testicular foreskin: 1
TUL:RCL	F: 11	7.0 (4.8–13.8)	67 (20–135)	None
PL	M: 2, F: 5	5.4 (2.3–9.9)	94 (48–133)	None

UUL, ureteroscopic urethral laser lithotripsy; UUBL, ureteroscopic urethral and bladder laser lithotripsy; UBL, ureteroscopic bladder laser lithotripsy; RCL, rigid cystoscope laser lithotripsy; PL, percutaneous laser lithotripsy.

### 3.3 UUBL cases

Three of 24 male dogs (12.5%) were selected for laser lithotripsy of the urethra and bladder using a flexible ureteroscope. The median operation time was 129 min (90–236 min), and body weight was 5.1 (4.5–5.2) kg (Table 1). One dog (33.3%) had six stones < 3 mm in diameter in the urethra, and two (66.6%) had 2–4 stones measuring 6–12 mm in diameter blocking the urethra. During the procedure, crushed stones in the urethra were returned to the bladder to be further fragmented and dispersed. The fragments were collected in a basket, and whenever possible, the patient was made to stand and urinate to help flush even the smallest fragments. In one dog (33.3%), the procedure was converted to PCCL because it was difficult to secure a clear view due to bleeding, and the surgery was prolonged. Of the dogs that underwent UUBL, two (66.6%) completed the procedure without conversion. In one, the ureteral stones were returned to the bladder, and because the bladder stones were small, no additional laser lithotripsy was required. This dog, however, developed intra-abdominal urine retention after surgery, necessitating the placement of a 6Fr balloon catheter. Of the three dogs that underwent UUBL, two (66.6%) required treatment with a balloon catheter, and the third did not have a catheter inserted and developed testicle swelling and perineal edema. Thus, all the dogs that underwent the procedure developed complications.

### 3.4 UBL cases

Laser lithotripsy was performed in the bladder using a flexible ureteroscope in 12/41 (29.2%) dogs, of which 11/24 (45.8%) were male dogs and 1/17 (5.8%) was female. The duration of laser lithotripsy was 103 (56–230) min, and the median body weight was 13.1 (3.0–49.2) kg (Table 1). Two dogs, a 3.0 kg male Yorkshire Terrier and a 4.0 kg mixed breed dog, had previously undergone perineal urethrostomy, allowing for laser lithotripsy via flexible ureteroscope insertion through the perineal urethrostomy site. The maximum diameter of the bladder stones was ≥3 mm but < 6 mm in 3/24 (12.5%) male dogs, ≥6 mm but < 12 mm in 5/24 (20.8%) male dogs, ≥12 mm in 4/24 (16.6%) male dogs, and 1/17 (5.8%) female dogs. A rigid cystoscope was initially inserted into a 38.7 kg Labrador Retriever. However, although it reached the bladder, it did not allow access to the stones; therefore, a flexible ureteroscope was used instead. The stones were successfully fragmented in 230 min and collected using a basket; as much as possible, they were expelled by standing the dog up and encouraging urination to aid in natural stone elimination. The success rate for UBL was 12 dogs (100%). Complications occurred in one of 11 male cases (9%), presenting as testicular swelling and perineal edema.

### 3.5 RCL cases

The median time for the 11/17 (64.7%) female dogs that underwent laser lithotripsy for bladder stones using a rigid cystoscope was 67 (20–135) min. The median body weight was 7.0 (4.8–13.8) kg (Table 1). In 2/11 (18.1%) dogs, the maximum diameter of the stones was between 3 and 6 mm. In 3/11 (27.2%), the stones measured between 6 and 12 mm, and in 5/11 (45.4%), the stone diameter ranged from 12 to 24 mm. Fragments were collected as much as possible by basket retrieval and, when possible, by pressure urination and bladder washing in a standing position. The success rate of RCL was 100%. No complications were detected in any of the cases, and the postoperative course was good.

### 3.6 PL cases

PL was performed in 7/41 (17%) dogs, 2/24 (8.3%) male dogs, and 5/17 (29.4%) female dogs. The median operation time was 94 (48–133) min, and body weight was 5.4 (2.3–9.9) kg. The maximum diameter of the stones in 5/7 (71.4%) of the dogs was 12–24 mm, and in 2/7 (28.6%), it was 24–30 mm. Additionally, three dogs were noted to have sand-like stones. All fragments were efficiently retrieved through the port by hydrodynamic irrigation and basket retrieval. The success rate of PL was 100%. No significant complications were observed in any of the cases, and the postoperative course was good.

### 3.7 Extracted stones and urine bacterial culture test

In males, 15 had calcium oxalate, four had cystine, two had struvite, and two had mixed stones, with one unexamined. In females, 11 had struvite, five had calcium oxalate, and one had mixed stones. If the main component of the stone was classified as ≥70%, the remainder was classified as mixed stone.

Urine bacterial culture was performed by collecting urine via bladder puncture or catheterization in dogs that could be safely punctured before stone fragmentation. We avoided collecting urine from dogs with difficulty urinating whenever possible. Of the 41 dogs that were examined (13 males, 18 females), 11 (six males, five females) tested positive for infection, and 20 (seven males, 13 females) tested negative for infection. Eight dogs had at least one antimicrobial agent identified as effective based on in-house susceptibility testing. In the remaining three dogs, in-house susceptibility testing revealed no effective antimicrobial agents; external testing identified *Pseudomonas aeruginosa, Staphylococcus pseudintermedius*, and a mixed growth of *Escherichia coli* and *Enterococcus faecium*.

### 3.8 Total complications and follow-up

Of the 41 dogs that underwent laser lithotripsy, 39 (95.1%) were discharged from the hospital approximately 3 h after surgery. Of the three dogs that underwent UUBL, two (66.6%) developed urine leakage from the bladder and were admitted to the hospital for treatment. Post-surgical complications within 1 week included perineal and testicular edema and inflammation in three uncastrated dogs (one each after UUL, UBL, and UUBL). These dogs experienced genital pain and pollakiuria, but not hematuria or dysuria. Resolution of the complications was achieved with analgesics and antibiotics within 1 week. Owing to excessive bleeding and poor visibility, despite this dog undergoing UUL, stone collection from the bladder was difficult, leading to a prolonged surgery time. The procedure was converted to PCCL. After surgery, this dog developed ascites, and a balloon catheter was placed in the bladder as a precaution, and the dog was hospitalized for 1 week. In a case of an uncastrated male dog undergoing UUBL, bladder leakage was suspected during postoperative bladder washing, as only a small amount of washing fluid was recovered. This dog had a balloon catheter in place for 3 days, had developed a urinary obstruction before hospitalization, and had undergone multiple bladder punctures at the referring hospital. Following the removal of the balloon catheter, the dog had no urination difficulties and was discharged after 1 week.

No dogs required further laser lithotripsy or surgical intervention at 1 month postoperatively. X-rays and abdominal ultrasound were performed 1–3 months after laser lithotripsy. Ten of 28 dogs demonstrated residual or recurrent sand-like uroliths. Two dogs experienced clinically relevant recurrence requiring surgical intervention: an uncastrated male (UUL) with calcium oxalate urethral obstruction and another uncastrated male (UBL) with cystine urolithiasis, both within 3 months, underwent perineal urethrostomy. A third male (UBL) required re-lithotripsy at 10 months but has been managed with regular bladder lavage. Postoperatively, a dietary supplement (Urina ST) and a prescription diet were recommended for urolithiasis prophylaxis. In March 2024, we followed up with all dog owners in this study via phone to check their progress over the past year. We were able to contact 38 of them. Of the dogs that required surgery or stone fragmentation during follow-up, only three underwent re-surgery within 3 months of the initial procedure.

## 4 Discussion

In this study, we verified that laser lithotripsy could be performed on all dogs via TUL or PL. One dog that had TUL (UUBL) applied had to be converted to PL due to heavy bleeding and difficulty securing a clear surgical view. However, laser lithotripsy remained highly effective and was successfully performed in the other dogs. Adams et al. reported that the success rate of TUL was 100% in female dogs, 86.7% in male dogs, and 92% overall. They also stated that TUL is not indicated in male dogs weighing < 5 kg, as a flexible ureteroscope cannot be inserted into their urethra (21).

However, in this study, laser lithotripsy was successfully performed in male dogs weighing < 5 kg who could not undergo TUL by either inserting a flexible ureteroscope into the perineal urethra or performing PL in dogs that had previously undergone perineal urethral fistula surgery. As the male urethra is longer than the female urethra, it is more difficult to maneuver a flexible ureteroscope. Additionally, the laser fiber that can be used in the channel is limited to 272 microns, resulting in a weaker lithotripsy force. When considering operation time, PL should be selected in cases where the stone is large, there are multiple stones, or the stone can be pushed into the bladder. Therefore, it is necessary to always be prepared to transition to other minimally invasive procedures, such as PCCL or PL, when required (11, 12, 15, 19). Females can be treated using rigid cystoscopes, which provide a larger field of view than flexible cystoscopes and are easier to operate with a higher success rate (21, 22). This may be due to rigid cystoscopes accommodating thicker, high-powered laser fibers (365 microns) or because female dogs have a straight urethra that is easier to maneuver than that of males and that stone fragments are efficiently collected. In contrast, in this study, one case (a 38.7 kg female Golden Retriever) required an alternative approach, as the rigid cystoscope used could not reach her bladder. In this case, 230 min were required for a flexible endoscope to fragment the stone to a size that could pass through the urethra. The main factors contributing to the long procedure time were the presence of two stones with a maximum diameter of 2.6 cm, the use of a flexible endoscope, which limited procedural efficiency, and the use of a thinner laser fiber (272 microns), which reduced lithotripsy power. These limitations could be addressed by using a slightly longer rigid cystoscope for large-breed female dogs, allowing improved access and enhanced lithotripsy efficiency.

One of the characteristics of holmium-YAG lasers is that they can be used safely with saline irrigation due to their high water-absorption rate. The laser's depth of tissue penetration is only 0.4 mm, and when the laser fiber is positioned 5.0 mm away, almost no tissue damage is detected.

When irradiated at a distance of ≤ 2.0 mm, the laser can achieve hemostasis and perform tissue resection, minimizing damage to the urethral and bladder walls, making it a safe option for veterinary patients. In this study, this procedure was conducted safely with few complications.

Previous reports have indicated that holmium-YAG lasers have a high surgical success rate. Additionally, sex, type of endoscope, and stone hardness were determined to significantly influence stone fragmentation time, with complications including testes edema in uncastrated males (21–23). This may be due to the difference in water pressure between the prostatic urethra and testes, as laser lithotripsy is always performed with continuous saline irrigation. Therefore, when performing UUL, UUBL, or UBL, the testes of uncastrated males may become edematous; owners must be informed about this potential complication before surgery. With techniques other than PL, sand-like fragments or small residual fragments that are smaller than the urethral diameter may remain in the bladder, making complete stone removal impossible. In a report by Lulich et al., it was suggested that fragments < 3 mm in diameter, which can pass through the urethra of dogs, may be naturally expelled, thus, additional treatment may not be necessary. No clear criteria exist for determining what fragment size in dogs should be classified as residual stones and whether they pose a risk of infection or worsening of urinary tract symptoms (25). Differences in stone composition were considered unlikely to have influenced the difficulty of fragmentation.

Laser lithotripsy complications include hypothermia, urethral perforation, and postoperative hematuria, which are associated with the use of large volumes of saline irrigation. These complications potentially occur due to bladder mucosal fragility instigated by pre-existing cystitis and the impact of stone fragments striking the mucosa during fragmentation, leading to localized bleeding (21). In a dog that underwent UUBL, significant intraoperative bleeding was observed to obscure the surgical field, necessitating conversion to PCCL. Considering that other studies have reported cases where conversion to conventional laparotomy was required, the ability to continue minimally invasive surgery in such cases may be beneficial. One male dog that underwent UUBL developed ascites during bladder irrigation following fragmentation, as detected by abdominal ultrasound. Bladder fragility was suspected to have been exacerbated by multiple prior bladder punctures, which were performed at another hospital due to complete urethral obstruction before referral. Further treatment was not required, as the placement of a 6Fr balloon catheter was sufficient to manage the condition.

In this study, two cases required repeat stone fragmentation within 1 year of surgery. In one case, a male dog that had undergone UBL developed recurrent calcium oxalate bladder stones. After the second fragmentation procedure, this dog was managed with regular bladder washes. Although millimeter-sized, sand-like stones remained, no further fragmentation was required for 74 months. In another case, a male Newfoundland with cystine stones that had undergone UBL developed recurrent cystine urolithiasis 2 months after the second laser lithotripsy, and the owner requested a perineal urethrostomy.

In this study, several dogs had previously undergone multiple bladder incision surgeries due to urinary tract stone disease. Considering that these patients had required repeated abdominal surgeries, this surgical method presents an attractive minimally invasive treatment option. In female dogs, laser lithotripsy is a highly effective treatment if a rigid cystoscope can be inserted. However, in male dogs, laser lithotripsy is more technically challenging, and anesthesia time is prolonged unless the urethral diameter, stone size, and stone quantity are carefully evaluated preoperatively (21, 22, 25). By incorporating the surgical techniques for PL and PCCL, minimally invasive surgery for lower urinary tract stones could be performed more effectively. In this study, the flexible ureteroscope was damaged six times during TUL, as the tip of the endoscope was damaged by stone fragments rebounding upon impact during fragmentation (22). Considering the high cost of repairs, the use of disposable flexible ureteroscopes may be necessary in the future.

This study has certain limitations. First, the small sample size. Second, as data were collected exclusively from our hospital, a multi-center study could not be conducted. Therefore, significant case selection bias may exist. Third, all surgeries were performed by a single surgeon with extensive experience in endoscopic procedures. No comparison was conducted on the impact to surgical outcomes between operators with different levels of expertise. In the future, prospective studies at multiple facilities will be necessary to further evaluate the safety and efficacy of these surgical techniques. Additionally, it will be important to establish guidelines for laser fragmentation criteria in dogs to standardize treatment approaches.

## 5 Conclusion

TUL is a highly minimally invasive procedure, but it presents technical challenges when performed on male dogs. However, PL can be an effective surgical procedure, and it can be applied regardless of stone size, stone number, patient weight, or sex.

## Data Availability

The original contributions presented in the study are included in the article/supplementary material, further inquiries can be directed to the corresponding author.
